# Preventive Aortic Stent Graft Implantation Prior to Thoracic Surgery: Early and Midterm Results

**DOI:** 10.3390/jcm13195694

**Published:** 2024-09-25

**Authors:** Olivia Lauk, Bianca Battilana, Didier Schneiter, Isabelle Schmitt-Opitz, Alexander Zimmermann, Benedikt Reutersberg

**Affiliations:** 1 Department of Thoracic Surgery, University Hospital Zurich, Rämistr. 100, 8091 Zurich, Switzerland; olivia.theisen-lauk@usz.ch (O.L.); bianca.battilana@usz.ch (B.B.); didier.schneiter@usz.ch (D.S.); isabelle.schmitt-opitz@usz.ch (I.S.-O.); 2 Department of Vascular Surgery, University Hospital Zurich, Rämistr. 100, 8091 Zurich, Switzerland; alexander.zimmermann@usz.ch

**Keywords:** descending thoracic aorta, lung cancer, endovascular surgery, thoracic surgery, TEVAR, aortic arch, supra-aortic debranching

## Abstract

**Background**: There is a paucity of data concerning the feasibility and value of thoracic aortic stent graft implantation (TEVAR) applications for removing tumors infiltrating the aortic wall. This analysis aimed to demonstrate the feasibility of TEVAR and monitor the perioperative risks of morbidity and mortality. Additionally, a literature review was performed. **Methods**: A retrospective data analysis was performed on patients who received TEVAR prior to thoracic malignancy resection between January 2010 and April 2024. The primary endpoint was technical success. **Results**: A total of 15 patients (median age: 67 years; range: 23–75; 66.7% female) received TEVAR prior to thoracic surgery of different tumor entities. In 80% of cases (n = 12), the proximal landing zone was in aortic zone 3. In three cases, the supra-aortic debranching of LSA and/or LCCA via bypass implantation or in situ laser fenestration was necessary. No postoperative endograft-related complications were observed. In eight patients, aortic wall infiltration was confirmed intraoperatively. In total, R0 resection was achieved in seven patients (46.7%). The 30-day mortality rate was 6.7% (n = 1). Technical success was achieved in all patients (100%), while procedural success was achieved in 80% due to incomplete tumor resection in three patients. **Conclusions**: To the best of our knowledge, this is the largest analysis to date that confirms the results of previous smaller studies. Aortic stent grafting prior to thoracic tumor resection allows for extensive resection while maintaining low morbidity and a low 30-day mortality risk.

## 1. Introduction

Tumor mass infiltration into the thoracic aorta, originating as a locally advanced tumor from the lung or as lung metastasis, can pose significant intraoperative risks, particularly bleeding complications due to injury to or erosion of the aortic wall [1,2,3,4,5,6,7]. Examining all the stages of lung carcinomas, T4 tumors appear infrequently, constituting approximately 4.4%. The occurrence of lung carcinomas infiltrating the aorta is even more uncommon, making up less than 1% of all T4 tumors [8,9]. These cases are further complicated by the fact that surgery of tumors invading the aorta could lead to incomplete resection margins and even tumor survival [10]. Complete resection may require aortic cross-clamping, possible prosthetic replacement, and extracorporeal circulation techniques to avoid fatal bleeding during surgery, with potentially life-threatening risks [11].

After the pioneering work by Volodos and colleagues in the early 1990s, who introduced the concept of utilizing stent grafts for the endovascular repair of abdominal and thoracic aneurysms, this less invasive approach has now established itself as the primary method for treating aortic pathologies [12]. The first case report on the off-label use of endografts in oncology was published in 2008 by Marulli et al. [13]. Since then, only a handful of studies have reported using TEVAR (thoracic endovascular aortic repair) to aid the en bloc resection of thoracic aortic wall-invading tumors [7,11,13,14,15,16,17,18,19,20,21,22,23]. In cases where the tumor mass is located near the origins of the supra-aortic branches, achieving a secure seal or sufficient safety margin may require additional open or endovascular debranching techniques. These procedures carry additional risks, such as stroke or spinal ischemia. However, limited data are currently available on these outcomes.

This analysis aimed to evaluate the feasibility of employing TEVAR prior to major thoracic cancer surgery, with a focus on the additional risks of perioperative morbidity and mortality.

## 2. Materials and Methods

All patients with suspected tumor mass infiltration of the aortic wall due to thoracic cancer treated with thoracic aortic stent graft implantation before tumor surgery at the University Hospital Zurich between January 2010 and April 2024 were retrospectively analyzed.

The data were collected from the clinical information system database at the University Hospital Zurich and input to an anonymized and password-protected data file (Excel Microsoft 2016). The clinical data included demographic characteristics, cardiovascular risk profiles, clinical presentation, preoperative and postoperative computed tomography angiography (CTA)-detected morphometric variables, and follow-up data. The institutional ethics committee approved the study protocol; written informed consent was obtained from all patients, or their enrolment was covered by paragraph 34 of the National Federal Human Research Act (BASEC Number 2021-02067). In addition, a literature review was conducted.

### 2.1. Inclusion and Exclusion Criteria

All patients with suspected tumor mass infiltration of the aortic wall due to thoracic malignancy and treated with thoracic aortic stent graft implantation before tumor surgery were included. Patients who declined further use of their data were excluded.

### 2.2. Preoperative Staging, Operation Planning, and TEVAR Technique

Preoperative assessment and staging included comprehensive, contrast-enhanced computed tomography (CT) of the chest and abdomen in most cases. Magnetic resonance imaging (MRI) was conducted in situations where there was uncertainty about cardiovascular infiltration or the presence of brain metastasis. Fluorodeoxyglucose-positron emission tomography (FDG-PET) was undertaken where there were doubts about the presence of metastatic lesions or for further clarification of tumor behavior. All the patients were discussed in an interdisciplinary tumor board prior to surgery. In cases where imaging revealed a suspected infiltration of the aortic wall, endovascular lining with TEVAR was planned prior to thoracic surgery.

TEVAR: Usually, femoral access is chosen under intubation anesthesia. After ultrasound-guided retrograde puncture, a large access sheet matching the prosthesis, measured using the preoperative CTA (maximum oversizing of 10%), is inserted via a stiff wire under fluoroscopic control. The corresponding prosthesis is inserted and released after angiography to visualize the supra-aortic branches and celiac trunk. The pathology length to be covered is determined using the vertebral bodies as landmarks to exceed the pathology proximally and distally by at least 2 cm. If this cannot be achieved because the supra-aortic vessels are close to the pathology, debranching must be performed to extend the landing zone, e.g., via the transposition or bypassing of the left subclavian artery.

### 2.3. Outcome Criteria

The primary outcome was technical success, defined as successful TEVAR without complications such as hemorrhage, stroke, endoleaks, or an inadequate overlap zone regarding the tumor mass (at least 2 cm proximal and distal of the tumor mass) evaluated on postoperative CT images.

The secondary outcome was successful TEVAR and the completion of the planned tumor mass resection without bleeding complications, also reported as procedural success. Additionally, the peri- and postoperative morbidity and mortality were analyzed.

### 2.4. Statistics

A descriptive data analysis was performed. Data are shown as percentages, medians, and ranges. The results of the study will be reported according to the STROBE guidelines for reporting observational studies [24] (completed checklist see Supplementary Materials). The statistical analysis was conducted using Excel 2016 and IBM SPSS Statistics Version: 29.0.0.0.

## 3. Results

### 3.1. Demographics

A total of 15 patients (median age: 67 years; range: 23–75; 66.7% female) were treated at our institution between January 2010 and April 2024. Table 1 and Table 2 present the demographics and comorbidities and the different tumor entities with the corresponding tumor stages, respectively.

### 3.2. Treatment

Ten patients were treated with neoadjuvant therapy, including chemotherapy (n = 6) or combined radio–chemotherapy (n = 4). In all patients, it was possible to implant the stent graft with sufficient overlap at the tumor mass level (Table 3). Thirteen of the fifteen patients were treated with a single stent graft, and only two patients received two prostheses to cover the adjacent tumor mass with a sufficient safety margin of at least 2 cm. Most patients (n = 10) received a TAG stent graft from W. L. Gore & Associates, Inc. (Newark, DE, USA) without bare springs. Five patients received stent grafts with bare springs: three Zenith alpha thoracic from Cook Medical (Bloomington, IN, USA) and two Evita (Jotec, Hechingen, Germany). In most cases (12 cases/80%), the proximal landing zone was in aortic arch zone 3. Extension into aortic arch zones 2 and 1 in two cases and one case, respectively, was necessary to have sufficient proximal coverage of the tumor mass. Supra-aortic vessels had to be debranched for this purpose. For zone 2, this was conducted in one case by placing an LSA bypass on the left common carotid artery (LCCA) and in another very recent case via laser in situ fenestration and bridging stent graft implantation. For the patient who required zone 1 debranching, a carotid–carotid–subclavian bypass had to be implanted, which led to LSA and LCCA debranching. This provided a sufficient landing zone to adequately cover the tumor mass. Vascular and thoracic surgeries were performed in a single joint operation in 2 patients and as two-stage procedure in 13 patients.

Six patients underwent a left- or right-sided thoracotomy in the fourth or fifth intercostal space. In three patients, access via hemi-clamshell was performed. Nine patients underwent an open approach (thoracotomy n = 6, semi-clamshell n = 3), four patients underwent robotic-assisted thoracoscopic surgery, and one patient had conventional, video-assisted thoracoscopic surgery. In one patient, thoracic surgery could not be performed because the esophageal tumor had progressed further under chemotherapy. Tumor resection was performed either by an anatomical or non-anatomical resection, including wedge resection (n = 2), lobectomies (n = 5), extrapleural pneumonectomies (n = 3), one completion pneumonectomy (n = 1), and one pure pleurectomy (n = 1). In two cases, additional pericardial (n = 3) and diaphragm (n = 1) resections were performed. Furthermore, para-aortal or mediastinal tumor resection (n = 5; n = 1 thymectomy) and one esophagectomy (n = 1) were achieved. Para-aortal resection refers to tumor resections attached to the aorta, without further details on the extent of the resection. In one case, the resection could not be completed due to an inoperable situs. The tumor extended too far centrally, with the infiltration of the ascending aorta and main trunk of the left pulmonary artery. Furthermore, the tumor extended to the carina in the left main bronchus area, which would have resulted in pneumonectomy, and this was not indicated for a bulky stage N2. One patient was operated on twice: the first via RATS and the second via an open approach with the rest-lobectomy of the left upper lobe. A third case only underwent the first step with TEVAR, and no further thoracic surgery was performed due to rapid progression and an advanced tumor burden.

### 3.3. Outcomes

**Figure 1:**
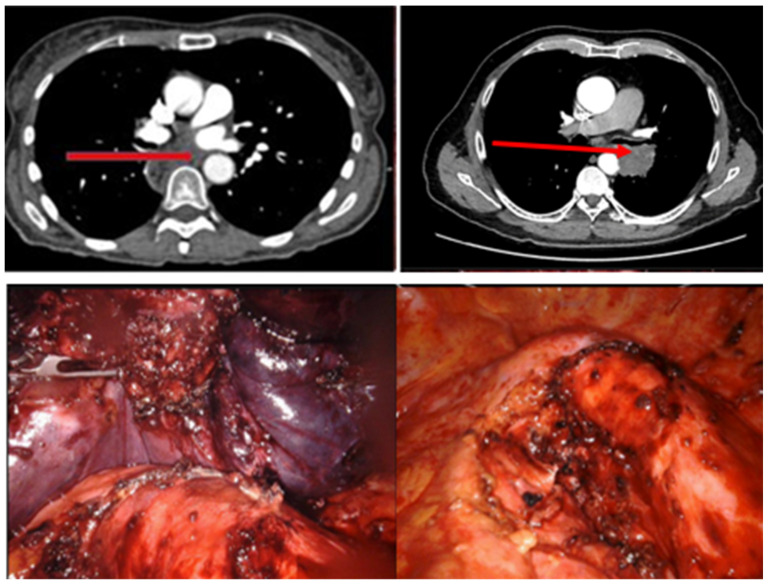
**Top**: Preoperative CT scan with the tumor mass in the posterior mediastinum with suspected aortic wall infiltration. **Bottom**: Intraoperative view of the partially resected tumor, including aortic wall.

No postoperative endograft-related morbidity was recorded, specifically, no spinal cord ischemia, stroke, postoperative bleeding, access site complications, dissection, or aortic rupture (Table 4). R0 resection was achieved in seven patients. In eight patients, aortic wall infiltration was confirmed intraoperatively, and a partial, non-circumferential resection of the aortic wall was necessary, resulting in R0 not being achieved in all cases (Table 4 and Figure 1).

A systematic lymph node dissection was performed regardless of the type of tumor resection. One patient underwent revision surgery 20 days after the initial thoracic surgery, during which an intrathoracic infection with additional old hematoma was cleared, and an intrathoracic vacuum dressing was placed. This was followed by an eventual vacuum dressing removal a few days later.

The median follow-up time was 15 months (range 0.7–93.5 months). The 30-day mortality rate was 6.7% (n = 1) due to respiratory failure, ARDS, and postoperative empyema. Within the 90-day period, a further two patients (day 49 and day 79) died of cancer-related causes (90-day mortality rate 20%, n = 3). Overall, 53% (n = 8) of the patients died during the observation period. Six of these were clearly attributable to the underlying tumor disease, although in two cases the cause remains unclear. However, a relationship to the TEVAR surgery appeared unlikely. No complication or mortality attributable to the TEVAR procedure was observed in any of the patients during follow-up. 

Ultimately, the primary outcome, technical success, was achieved in all the patients (100%). The secondary outcome, procedural success, was achieved in 80% of the patients as tumor resection could not be completed in three patients. Two patients developed postoperative respiratory complications that were unrelated to the aortic tumor resection and, thus, were not included as procedural failure.

## 4. Discussion

This analysis was able to demonstrate the feasibility of TEVAR without increased risks of perioperative morbidity and mortality. Depending on the tumor infiltration location, in some cases, the stent graft was implanted from the descending aorta into the aortic arch, including supra-aortic vessels, to expand the proximal landing zone by adjunct (e.g., carotid–carotid–subclavian bypass/transposition) or endovascular (e.g., in situ fenestration, etc.) procedures. The preventive measures of these techniques remain uncommon in treating advanced thoracic tumors and are still not widely adopted, contributing to the paucity of available data. Stent graft implantation and proximal landing zone extension with additional debranching operations were proven safe and feasible, with a 100% technical success rate, addressing the issue of increased risks of perioperative morbidity and mortality.

To the best of our knowledge, this is the largest analysis to date. It confirms the results of the previous studies collated from our literature review. None of the published studies reported on more than 10 patients (Table 5) [7,11,14,15,16,17,18,19,20,21,22,23].

Sato et al. reported one complication in stent implantation from a dissection of the external iliac artery, although it caused no further harm [21]. Aortic stent grafting, prior to thoracic tumor resection, has emerged as a crucial technique that enables extensive resection while ensuring low morbidity and a remarkably low 30-day mortality risk, also shown in our cohort. This study supports the findings of earlier studies, emphasizing the efficacy and safety of this approach. No postoperative endograft-related morbidity, spinal cord ischemia, stroke, postoperative bleeding, access site complications, dissection, or aortic rupture were observed. By employing aortic stent grafting, surgeons can effectively navigate the complexities of thoracic tumor resections, offering patients an improved prognosis and quality of life. The positive outcomes observed in this comprehensive analysis further validate the growing recognition of aortic stent grafting as a viable option for surgical interventions in thoracic oncology.

Major lung resection is associated with increased risks of peri- and postoperative morbidity and mortality. In a recently published, large, retrospective analysis of the European Society of Thoracic Surgeons database on 25,983 patients, the incidence of cardiopulmonary complications was analyzed. Stratified by risk probability, in the moderate-risk group, the results were as follows: 21% after lobectomy (294/1435), 29% after bilobectomy (33/112), 22% after pneumonectomy (72/333), and 16% after segmentectomy (22/136) (analysis of variance test, *p* = 0.07) [25]. Morbidity for major lung resection varies between 27% and 44%, according to the literature, with a mortality rate between 1.4% and 5.4% [26,27] (15, 16). In our study, only one patient died within 30 days due to respiratory failure and ARDS combined with postoperative empyema.

Like Sasahara et al. and D’Andrilli et al., we recommend that thoracic surgical resection of the oncological findings should take place no longer than 7 to 10 days after stent prothesis implantation, as the perivascular inflammatory reaction caused by stent implantation makes it significantly more difficult to perform sufficient perivascular resections. However, in our view, there is no contraindication to performing both procedures within one operation [7,28].

### Limitations

The retrospective nature of this analysis is a limitation itself. Even though, to the best of our knowledge, this is the largest study, the data must be viewed with caution due to the small sample size and cannot be generalized. Conducting larger-scale studies would be essential to provide a conclusive recommendation. However, these instances are relatively rare, making it challenging to conduct a comprehensive analysis. The cohort’s size is insufficient to make a definitive statement. Nonetheless, this current analysis can serve as valuable data for potential inclusion in meta-analyses or reviews, offering better insights and treatment recommendations for these exceptional cases characterized by advanced tumor diseases. Larger multi-center studies with a prospective investigation are necessary to provide more robust conclusions.

## 5. Conclusions

In the seemingly largest analysis to date, the findings of previous studies were validated. Aortic stent grafting before thoracic tumor resection enables extensive resections while ensuring low morbidity and a low risk of 30-day mortality.

## Figures and Tables

**Table 1:** Demographics, tumor stage, and comorbidities (N = 15).

		N	Percentage
Female		10	66.7%
Age at surgery (median and range)		67	(23–75)
Preoperative tumor stage	T1 *	1	6.7%
	T2 **	1	6.7%
	T3	4	26.7%
	T4	6	40%
	Missing	3	20%
Smoking		8	53.3%
Chronic kidney disease		2	13.3%
Hemodialysis		0	0.0%
Arterial hypertension		5	33.3%
Coronary heart disease		4	26.7%
Congenital heart disease		0	0.0%
Diabetes mellitus		3	20.0%
Dyslipidemia		3	20.0%
COPD		4	26.7%
PAD		0	0.0%

If not stated otherwise, numbers are given as numbers and percentages. COPD = chronic obstructive pulmonary disease; PAD = peripheral arterial disease. * The patient with a T1-stage tumor presented a broad base of contact with the aortic wall but no confirmed infiltration of the aorta in the preoperative MRI. As a precaution, TEVAR was still performed. ** The patient with a T2-stage tumor presented pulmonary metastasis of an adenoid cystic carcinoma of the submandibular gland, and the staging refers to the primary diagnosis.

**Table 2:** Tumor entities with the corresponding tumor stages.

Case	Tumor	T	N	M	Surgery
1	Metastases	NA	NA	NA	Extrapleural pneumonectomy and pericardial resection
2	Pleural mesothelioma	4	1	0	Pleurectomy, lower lobe wedge resection
3	NSCLC (squamous cell carcinoma)	4	0	0	Extrapleural pneumonectomy and pericardial resection
4	NSCLC (adenocarcinoma)	1	2	0	Parital tumordebulking and para-aortic resection
5	Malignant peripheral nerve sheath tumor	NA	NA	NA	Para-aortic tumor resection
6	Esophageal cancer	4	1	0	No surgery
7	NSCLC (squamous cell carcinoma)	4	2	1	Left lower lobe lobectomy and para-aortic resection
8	Metastasis	NA	NA	NA	Extrapleural pneumonectomy and para-aortic and diaphragm resection
9	NSCLC (adenocarcinoma)	3	0	1	Left lower lobe lobectomy
10	Esophageal carcinoma	4	1	0	Left lower lobe lobectomy and esophagectomy
11	Esophageal carcinoma	3	1	0	Lymph node resection in the tracheobronchial angle
12	Esophageal carcinoma	3	1	0	Para-aortic tumor resection
13	Thymus carcinoma	3	0	0	Mediastinal tumor resection, wedge resection left lower and upper lobe followed by completion lobectomy left upper lobe, pericardial resection,
14	NSCLC (squamous cell carcinoma)	2	2	0	Completion pneumonectomy left
15	NSCLC (adenocarcinoma)	4	0	1	Left upper lobe lobectomy

Esophageal carcinoma: squamous cell carcinoma of the esophagus; NA: not applicable.

**Table 3:** Treatment (N = 15).

		N	Percentage
Neoadjuvant Therapy		10	66.7%
Neoadjuvant therapy treatments	CTX alone	6	40.0%
	RTX alone	0	0.0%
	RTX and CTX	4	26.7%
Radiotherapy dose (Gy)	40–60	2	13.3%
	60–80	2	13.3%
	>80	0	0.0%
Radiotherapy duration	1–5 days	0	0.0%
	5–7 days	0	0.0%
	7–14 days	1	6.7%
	>14 days	3	20.0%
Chemotherapy substances	Platinum based	8	53.5%
	Immunotherapy	1	6.7%
	TKI	0	0.0%
	Other	1	6.7%
Cycles applied	1–3	7	46.7%
	4–6	3	20.0%
	>6	0	0.0%
Vascular surgery (TEVAR)			
Proximal landing zone	Z0	0	0%
	Z1	1	6.7%
	Z2	2	13.3%
	Z3	12	80%
	Z4	0	0%
Debranching technique and extent	LSA bypass/transposition	1	6.7%
	LCCA and LSA bypass	1	6.7%
	Brachiocephalic trunk, LCCA and LSA bypass	0	0.0%
	Laser in situ fenestration LSA	1	6.7%
	Parallel graft	0	0.0%
Thoracic tumor resection			
Open approach	Posterolateral thoracotomy	1	6.7%
	Antero-lateral thoracotomy	5	33.3%
	Median sternotomy	0	0.0%
	Hemi-clamshell	3	20.0%
	Other	5	33.3%

CTX—chemotherapy; Gy—gray; LCCA—left common carotid artery; LSA—left subclavian artery; RTX—radiotherapy; TKI—tyrosine kinase inhibitor.

**Table 4:** Outcomes.

		N	Percentage
Tumor resection status	R0	7	46.7%
	R1	2	13.3%
	R2	4	26.7%
	Palliative/tumor debulking	1	6.7%
Aortal resection (diameter)	None	6	40.0%
	<1 cm	2	13.3%
	1–5 cm	3	20.0%
	5–10 cm	3	20.0%
	>10 cm	0	0.0%
Localization of R1/R2 (multiple possible)	Paraaortic	5	33.3%
	Esophagus	0	0.0%
	Paravertebral	1	6.7%
	Mediastinal	3	20%
	Chest wall	2	13.3%
Aortic rupture		0	0.0%
Endoleak		0	0.0%
Myocardial infarction		0	0.0%
Stroke		0	0.0%
Paraplegia		0	0.0%
Respiratory failure after vascular intervention		0	0.0%
Intermittent dialysis		1	6.7%
Bleeding		0	0%
Respiratory insufficiency after thoracic intervention		1	6.7%
ARDS		1	6.7%
Empyema		2	13.3%
Bronchial insufficiency		0	0.0%
Overall mortality		8	53.3%
30-day mortality		1	6.7%
Causes of death	Aortic-related	0	0.0%
	Cancer-related	6	40.0%
	Unknown	2	13.3%

ARDS—acute respiratory distress syndrome. Para-aortal resection refers to tumor resections attached to the aorta, without further details on the extent of the resection. R—residual tumor.

**Table 5:** Literature review.

Study	Year Published	Total Endovascular-Treated Patients	Age (Mean)	Days between Stenting and Surgery	Endograft-Related Complication	30-Day Mortality	Mean FU (Months)
Roche-Nagle et al. [14]	2009	2	43 y, 52 y	NS/5	0	NS	NS
Berna et al. [15]	2011	1	59	0	0	0	23
Collaud et al. [16]	2014	5	52 (median)	1–27	0	NS	39
Otani et al. [18]	2016	1	79	20	0	0	30
Mody et al. [19]	2016	1	71	10	0	0	NS
Walgram et al. [20]	2018	3	58	0–28	0	NS	24
Marulli et al. [11]	2015	9	61	NS	0	NS	NS
Dejima et al. [17]	2016	1	82				
Sato et al. [21]	2019	6	66	NS	1	NS	17.6 (median)
Di Tommaso et al. [22]	2023	5	58	NS	0	NS	21
Danial et al. [23]	2023	9	62	7 (1–18)	0	0	25 (median)
D’Andrilli et al. [7]	2023	7	67	NS	0	1 (14.3%)	38 (median)

NS = not specified.

## Data Availability

The data underlying this article will be shared upon reasonable request to the corresponding author.

## Supplementary Materials

The following supporting information can be downloaded at: https://www.mdpi.com/article/10.3390/jcm13195694/s1, Completed STROBE checklist.
