# Supplementary material for: Preventive Aortic Stent Graft Implantation Prior to Thoracic Surgery: Early and Midterm Results
Source: J Clin Med. 2024 Sep 25;13(19):5694. doi: 10.3390/jcm13195694 (PMC11477123; doi:10.3390/jcm13195694)
Supplement: Supplementary file 1 [file jcm-13-05694-s001.zip › jcm-3179564-supplementary.pdf]

STROBE Checklist for the manuscript titled "Preventive Aortic Stent Graft Implantation Prior to Thoracic Surgery: Early and Midterm Results":

Title and Abstract

1. **Title**: The title should clearly indicate that the study is observational.
   - **Completed**: Yes
   - **Title**: Preventive aortic stent graft implantation prior to thoracic surgery: Early and midterm results
2. **Abstract**: Provide a structured abstract including Background, Methods, Results, and Conclusion.
   - **Completed**: Yes
   - **Abstract**: Provided in the manuscript

Introduction

3. **Background/Rationale**: Explain the scientific background and rationale for the study.
   - **Completed**: Yes
   - **Details**: Background provided in Introduction section explaining the need for TEVAR before thoracic surgery and the gap in literature.
4. **Objectives**: State the specific objectives or hypotheses.
   - **Completed**: Yes
   - **Details**: Objectives clearly outlined as evaluating feasibility, perioperative risk, and a literature review.

Methods

5. **Study Design**: Describe the study design (e.g., cohort, case-control).
   - **Completed**: Yes
   - **Details**: Retrospective data analysis
6. **Setting**: Describe the setting, locations, and relevant dates.
   - **Completed**: Yes
   - **Details**: University Hospital Zurich, January 2010 to April 2024
7. **Participants**: Describe the eligibility criteria, settings, and locations.
   - **Completed**: Yes
   - **Details**: Patients with tumor mass infiltration of the aortic wall who received TEVAR before tumor surgery
8. **Variables**: Define all variables, including outcome variables.
   - **Completed**: Yes
   - **Details**: Primary outcome: technical success; Secondary outcome: procedural success and perioperative morbidity/mortality
9. **Data Sources/Measurement**: Describe methods of data collection and sources.
   - **Completed**: Yes
   - **Details**: Clinical information system database, anonymized data file
10. **Bias**: Describe any potential sources of bias.
    - **Completed**: Yes
    - **Details**: Retrospective nature of the study and small sample size
11. **Study Size**: Explain how the sample size was determined.
    - **Completed**: Yes
    - **Details**: Sample size not determined statistically; limited by the number of available cases

12. **Quantitative Variables**: Define quantitative variables and describe any methods used to analyze them.
    - **Completed**: Yes
    - **Details**: Demographic and clinical data analyzed descriptively
13. **Statistical Methods**: Describe statistical methods used to analyze the data.
    - **Completed**: Yes
    - **Details**: Descriptive analysis, percentages, median, range; statistical software used: Excel and IBM SPSS

Results

14. **Participants**: Give the number of participants at each stage of the study.
    - **Completed**: Yes
    - **Details**: 15 patients included, details provided in Results section
15. **Descriptive Data**: Provide descriptive data for each group.
    - **Completed**: Yes
    - **Details**: Tables with demographic data, tumor entities, and treatment
16. **Outcome Data**: Report numbers of outcome events or summary measures.
    - **Completed**: Yes
    - **Details**: Outcomes including technical success, procedural success, complications, and mortality rates
17. **Main Results**: Provide the main results with confidence intervals if applicable.
    - **Completed**: Yes
    - **Details**: Results include technical success (100%), procedural success (80%), 30-day mortality rate (6.7%)
18. **Other Analyses**: Report any other analyses performed (e.g., subgroups).
    - **Completed**: Yes
    - **Details**: Analysis of tumor resection status and complications

Discussion

19. **Key Results**: Summarize key results with reference to objectives.
    - **Completed**: Yes
    - **Details**: Summarized feasibility and safety of TEVAR, comparison to literature
20. **Limitations**: Discuss limitations of the study.
    - **Completed**: Yes
    - **Details**: Small sample size, retrospective nature, limitations of generalizability
21. **Interpretation**: Interpret results with respect to hypotheses or objectives.
    - **Completed**: Yes
    - **Details**: Confirmed feasibility of TEVAR with low morbidity and mortality
22. **Generalizability**: Discuss generalizability of findings.
    - **Completed**: Yes
    - **Details**: Limitations in generalizability due to small sample size and retrospective design

Other Information

23. **Funding**: Describe funding sources.
    - **Completed**: Yes
    - **Details**: No external funding
24. **Conflict of Interest**: Declare any conflicts of interest.
    - **Completed**: Yes

- **Details**: The authors declared the following potential conflict of interest concerning publication of this article's research, authorship, and/or publication: Benedikt Reutersberg is a proctor for Terumo Aortic and Cook Medical. Alexander Zimmermann is a proctor for Cryolife/JotecArtivion, Terumo Aortic, Cook Medical, Lombard/Endovastec/Microport and iVascular. He is a member of an Advisory Board for Medtronic, Artivion, and iVascular. Isabelle Opitz declares the fol-lowing potential conflicts of interest: Roche (Institutional Grant and Speakers Bureau), Astra-Zeneca (Advisory Board and Speakers Bureau), MSD (Advisory Board), BMS (Advisory Board), Medtronic (Institutional Grant and Advisory Board), Intuitive (Proctorship).

25. **Ethical Approval**: State whether ethical approval was obtained.
    - **Completed**: Yes
    - **Details**: Approved by the institutional ethics committee, informed consent obtained
26. **Author Contributions**: Detail contributions of each author.
    - **Completed**: Yes
    - **Details**: Provided in the Author Contributions section
